# Risk Association, Linkage Disequilibrium, and Haplotype Analyses of β-Like Globin Gene Polymorphisms with Malaria Risk in the Sabah Population of Malaysian Borneo

**DOI:** 10.3390/genes13071229

**Published:** 2022-07-11

**Authors:** Eric Tzyy Jiann Chong, Lucky Poh Wah Goh, Ho Jin Yap, Eric Wei Choong Yong, Ping-Chin Lee

**Affiliations:** 1 Biotechnology Research Institute, Universiti Malaysia Sabah, Jalan UMS, Kota Kinabalu 88400, Sabah, Malaysia; eric_ctj@ums.edu.my; 2 Faculty of Science and Natural Resources, Universiti Malaysia Sabah, Jalan UMS, Kota Kinabalu 88400, Sabah, Malaysia; luckygoh@ums.edu.my (L.P.W.G.); yaphojin@hotmail.com (H.J.Y.); eric_yong11@hotmail.com (E.W.C.Y.)

**Keywords:** β-like globin gene, malaria, Malaysian Borneo, Sabah population, single nucleotide polymorphism

## Abstract

Single nucleotide polymorphisms (SNPs) in the β-like globin gene of the human hosts to the risk of malaria are unclear. Therefore, this study investigates these associations in the Sabah population, with a high incidence of malaria cases. In brief, DNA was extracted from 188 post-diagnostic blood samples infected with *Plasmodium* parasites and 170 healthy controls without a history of malaria. Genotyping of the β-like globin C-158T, G79A, C16G, and C-551T SNPs was performed using a polymerase chain reaction-restriction fragment length polymorphism approach. Risk association, linkage disequilibrium (LD), and haplotype analyses of these SNPs were assessed. This study found that the variant allele in the C-158T and C16G SNPs were protective against malaria infections by 0.5-fold, while the variant allele in the G79A SNP had a 6-fold increased risk of malaria infection. No SNP combination was in perfect LD, but several haplotypes (CGCC, CGCT, and CGGC) were identified to link with different correlation levels of malaria risk in the population. In conclusion, the C-158T, G79A, and C16G SNPs in the β-like globin gene are associated with the risk of malaria. The haplotypes (CGCC, CGCT, and CGGC) identified in this study could serve as biomarkers to estimate malaria risk in the population. This study provides essential data for the design of malaria control and management strategies.

## 1. Introduction

Malaria is a zoonotic disease caused by *Plasmodium* parasites transmitted by the *Anopheles* mosquitoes’ bites. Five *Plasmodium* species have been identified to infect humans, including *Plasmodium falciparum*, *Plasmodium vivax*, *Plasmodium ovale*, *Plasmodium malariae*, and *Plasmodium knowlesi*. In 2020, malaria cases were estimated to increase to 241 million globally [1]. Although malaria-related deaths have steadily decreased over the last 20 years, they increased by 12% in 2020 compared to 2019, resulting in a mortality rate of 15 deaths per 100,000 population [1]. The increase in malaria deaths recently is linked to the reducing access to health care services ranging from relevant diagnostic and clinical aspects due to the COVID-19 pandemic, especially in the African regions [2]. Since COVID-19 is still outspread endlessly around the globe, it is estimated that malaria deaths will continuously accelerate in the next few years.

Malaysia is also one of the countries significantly impacted by the malaria disease. Approximately 1.3 million of the Malaysian population are at risk of being infected by the *Plasmodium* parasites, and about 10,000 of them eventually live in active foci areas [1]. A recent study revealed that Sabah, Malaysian Borneo, has contributed to 43.3% of all malaria cases in Malaysia [3]. The fatal *P. knowlesi* is the most commonly reported species in Sabah. Many studies have been conducted to understand better the effects of this *Plasmodium* species in Sabah, including risk factors for infection with *P. knowlesi* and the genetic diversity of *P. knowlesi* [3,4,5,6].

Variants in a human gene are regularly associated with the risk of certain diseases. For example, the C-158T (rs7482144) single nucleotide polymorphism (SNP) in the 5′ region of the hemoglobin subunit gamma 2 gene is linked with higher fetal hemoglobin levels, sickle cell anemia, and thalassemia [7,8]. Interestingly, another SNP in the 5′ region of the β-globin gene, the C-551T (rs number is not available), is found as a silencer for the gene’s transcription and is associated with a thalassemic phenotype [9]. Furthermore, earlier research has linked the C16G (rs10768683) and G79A (rs33950507) SNPs in the globin gene to modifications in erythrocyte structure and thalassemia illness [10,11,12]. These SNPs are related to the function or structure of the red blood cells. Therefore, it is interesting to study whether they have malaria protection effects since SNPs in the β-like globin gene are most frequently related to malaria disease in several case–control or genome-wide association studies [13,14,15]. These data also indicate that SNPs in the β-like globin gene have a great potential in predicting the risk of malaria disease in human populations.

Sabah, a state with many rural areas and a hotspot for malaria transmissions, reported the highest malaria incidences from 2013 to 2017 compared to Sarawak and Peninsular Malaysia [3]. SNP association in the β-like globin gene with malaria risk in the Sabah population of Malaysian Borneo remains scarce. Thus, this study aims to determine the association, linkage disequilibrium (LD), and haplotype of the C-158T, G79A, C16G, and C-551T SNPs in the β-like globin gene with the risk of malaria in Sabah population of Malaysian Borneo.

## 2. Materials and Methods

### 2.1. Blood Samples Collection and DNA Extraction

A total of 188 post-diagnostic blood samples infected with *Plasmodium* parasites were collected from the Public Health Laboratory, Kota Kinabalu, Sabah. Among these samples, 134 blood samples were infected with *P. knowlesi*, 28 were infected with *P. falciparum*, and 26 were infected with *P. vivax*, as molecularly validated previously using the PlasmoNex^TM^ multiplex assay [16]. Besides that, 170 blood samples were randomly collected from healthy volunteers living within the Kota Kinabalu areas and without a medical history of malaria for this study. The inclusion criteria for this study were: (i) the subjects were able to donate 3 mL of their blood samples, and (ii) the subjects were able to provide written consent for the study. On the other hand, the exclusion criteria were: (i) the cases were not infected with *Plasmodium* parasite, and (ii) the controls were not living within the Kota Kinabalu areas and with a history of malaria infection. The genomic DNA was extracted from these blood samples using a previously described method [17]. The study was conducted following the Declaration of Helsinki, and ethical approval was obtained from the UMMC Medical Research & Ethics Committee (reference no.: 709.1).

### 2.2. Polymerase Chain Reaction-Restriction Fragment Length Polymorphism (PCR-RFLP)

A polymerase chain reaction (PCR) master mix consisting of 100 ng of DNA template, 1X of *GoTaq*^®^ Flexi Buffer (Promega, Madison, WI, USA), 1 unit of *GoTaq*^®^ Flexi DNA polymerase (Promega, Madison, WI, USA), 1.5 mM of MgCl_2_ solution, 0.2 mM of dNTP mixture, and 0.2 µM of both forward and reverse primers (Table 1) was prepared separately for each of the SNPs. The mixture was topped-up with sterile distilled water (sdH_2_O) until a final volume of 20 µL. The PCR conditions were set as follows: initial denaturation at 94 °C for 4 min; amplification of 35 cycles at 94 °C for 30 s, annealing temperature according to different primer sets (Table 1) for 30 s, and 72 °C for 45 s; a final extension at 72 °C for 4 min. The PCR products were electrophorized in 2% agarose gel stained with ethidium bromide.

A restriction fragment length polymorphism mixture consisting of 4 µL of PCR products, 2.5 units of restriction enzymes (New England Biolabs, Ipswich, MA, USA) according to the respective SNPs (Table 1), and 1X of CutSmart^TM^ Buffer (New England Biolabs, Ipswich, MA, USA) was prepared. The mixture was topped-up with sdH_2_O to a final volume of 15 µL and incubated overnight at 37 °C. The digested products were analyzed in 2–3% agarose gel stained with ethidium bromide (Figure S1), and the genotype for each SNP was recorded.

### 2.3. Statistical Analyses

The odds ratio (ORs) and 95% confidence interval (CI) were calculated using the SPSS software ver. 22.0 (IBM, Armonk, NY, USA). The LD and haplotype analyses of the SNPs were determined using the SHEsis software [21]. A *p*-value less than 0.05 was considered statistically significant.

## 3. Results

### 3.1. Association of the β-Like Globin SNPs with the Risk of Malaria

Our findings revealed that the T variant allele in the C-158T SNP was associated with a lower risk of malaria infection in general (OR = 0.48, 95% CI = 0.25–0.92, *p* = 0.027) and a lower risk of infection by *P. knowlesi* (OR = 0.45, 95% CI = 0.21–0.95, *p* = 0.035) (Table 2). On the other hand, the presence of the variant A allele in the G79A SNP was associated with a 6-fold increased risk of *P. falciparum* infection (OR = 6.36, 95% CI = 1.25–32.33, *p* = 0.026) (Table 3). Subjects who inherited a variant G allele for the C16G SNP had a 0.5-fold lower risk of *P. vivax* infection (OR = 0.46, 95% CI = 0.24–0.87, *p* = 0.017) (Table 4). There was no evidence of a link between C-551T SNP and the risk of malaria (Table 5).

### 3.2. LD Analysis of the β-Like Globin SNPs

**Figure 1:**
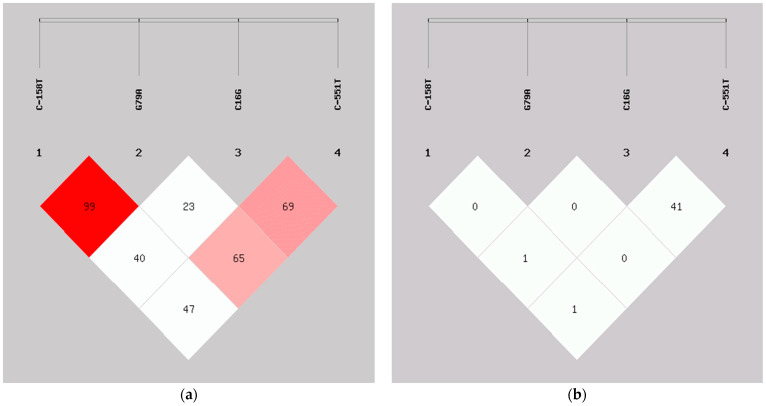
Degree of linkage disequilibrium of the β-like globin SNPs. (**a**) Values in the LD blocks indicated the D’ in percentage; (**b**) Values in the LD blocks represented the r^2^ in percentage.

The LD analysis showed that the C-158T and G79A SNPs combination was in a complete LD (D’ = 1.000) but with an extremely low r^2^ value (r^2^ = 0.001) (Table 6 and Figure 1), indicating these SNPs combination is not in a perfect LD. Nevertheless, other SNP combinations (C-158T and C16G, C-158T and C-551T, G79A and C16G, G79A and C-551T, and C16G and C-551T) were considered not in LD with moderately low D’ (<70%) and r^2^ (<50%) values.

### 3.3. Haplotype Analysis of the β-Like Globin SNPs with the Risk of Malaria

Table 7 represents the haplotype analysis based on the physical marker order: C-158T, G79A, C16G, and C-551T. The haplotype CGCC showed a 2.5-fold increased risk of malaria infections (OR = 2.51, 95% CI = 1.84–3.42, *p* < 0.001), whereas the haplotypes CGCT (OR = 0.17, 95% CI = 0.09–0.31, *p* < 0.001) and CGGC (OR = 0.07, 95% CI = 0.03–0.20, *p* < 0.001) were protective against the risk of malaria infection. Although the haplotypes CGGT and TGGT appeared to increase the risk of malaria infection, they were not statistically significant (*p* > 0.05).

## 4. Discussion

Sabah, Malaysian Borneo, had the highest malaria cases and deaths among all states in Malaysia from 2013 to 2017 [3]. Hence, determining the association of genetic polymorphism with the risk of malaria incidence with respect to its *Plasmodium* species can provide useful information for public health officials to design interventions for malaria cases, aligning with the vision of World Health Organization and the global malaria community in creating a world free of malaria. Several SNPs in the β-like globin gene have been reported to have different malaria susceptibility effects. This study investigated the association of the β-like globin C-158T, G79A, C16G, and C-551T SNPs with the risk of malaria in the Sabah population of Malaysian Borneo.

The C-158T SNP is located upstream of the γ-globin gene, which directly affects the production of hemoglobin F (HbF) [22]. This is correlated with the present study, where individuals who inherited a variant T allele in this SNP were significantly associated with a reduced risk of malaria, especially in *P. knowlesi* infection. Similarly, a pediatric malaria study reported that the spread of the *Plasmodium* parasite was retarded by HbF-containing red blood cells [23]. This suggests that the C-158T polymorphism might play a critical role in the variation of HbF levels that influence the protective risk effect against malaria. However, this study solely focuses on the genetic factor with the malaria risk, and the HbF levels of the subjects are not available.

Besides that, the G79A SNP is a mutation at codon 26 of the β-globin gene, which causes the hemoglobin E (HbE) variant [24]. This study observed that the variant A allele of this SNP was associated with a 6-fold increased risk of *P. falciparum* infection. Interestingly, a previous study has empirically shown that the variant allele created an alternative splicing site, which led to the production of a mutated beta-chain and is associated with a blood disorder, the HbE beta-thalassemia [25]. Despite reports suggesting people with blood abnormalities such as thalassemia can neutralize *Plasmodium* infection [26], the results of this investigation with the G79A polymorphism showed otherwise. The prevalence of thalassemia is high in Sabah, Malaysian Borneo [27]. As a result, the association between blood diseases and *Plasmodium* infection protection should be further explored.

The C16G SNP is located in the splicing region of the β-globin gene, and in silico studies revealed that the variant G allele could contain two possible branch sites that might induce a truncated protein [28,29], resulting in lower hemoglobin concentration. Malaria spreads by infecting the hemoglobin, and therefore a lower hemoglobin level logically results in a lower risk of malaria infection. This clearly explains that individuals who carried the variant GG genotype confer a protective risk effect against *P. vivax* infection by 0.5-fold, as observed in the present study. However, a recent study reported that infants with lower hemoglobin levels are not protected against malaria infection in a Papua New Guinean population [30]. Since the present study only involved adults and the hemoglobin level of the subjects is not assessed, further studies that investigate the risk of malaria in relationship with age differences and hemoglobin levels would be important for a better understanding of this complex interaction.

This study did not observe a significant association between the C-551T polymorphism and malaria risk. Despite its insignificant association, a correlation has been observed between the genotype distribution and malaria incidence. For instance, the Indian population has the highest C-551T polymorphism (79%), whereas the Greece population has the lowest C-551T polymorphism (37%) [31]. Simultaneously, India has a high malaria incidence, while malaria was eradicated in Greece in 1974 [1,32]. This might suggest an occurrence of genetic drift as the malaria incidence is eliminated in populations with a low percentage of C-551T polymorphism. However, the data should be carefully interpreted, since other factors, such as the geographical differences and the prevalence of *Plasmodium* species and *Anopheles* mosquitoes, have to be mutually considered.

A recent genome-wide association study on malaria parasites reported that LD and large haplotype blocks from 3.6 kb to 14.0 kb at chromosome 8 of *P. vivax* isolates were associated with different malaria transmission rates [33]. Therefore, it is interesting to study whether LD and haplotype of SNPs in the β-like globin gene of the human hosts are associated with the risk of malaria. Despite the lack of a perfect LD, association analysis based on the haplotype of this study indicated three significant haplotypes (CGCC, CGCT, and CGGC) with varying levels of correlation to malaria risk. These haplotypes could be used as biomarkers in the Sabah population to estimate malaria risk.

One of the limitations of this study is that the present study only focuses on the genetic aspect, and the hemoglobin levels of the human hosts were not assessed. Therefore, the interplay between the SNPs in the β-like globin gene and hemoglobin levels to the risk of malaria infection could not be estimated. Future studies should consider this limitation in their study designs.

## 5. Conclusions

In conclusion, this is the first study to investigate the association of the β-like globin SNPs with the risk of malaria in the Sabah population of Malaysian Borneo. This study suggests that the C-158T and C16G SNPs are protective against malaria infection, while the G79A SNP is an increased risk factor for malaria infection. Several haplotypes (CGCC, CGCT, and CGGC) with different correlation levels to malaria risk were identified, and they could serve as biomarkers for malaria risk estimation in the population. The data of this study could be essential to understanding the hosts’ immune responses to malaria susceptibility and the development of antimalarial drugs to treat malaria.

## Figures and Tables

**Table 1:** Primers used and PCR-RFLP conditions for the SNPs in this study.

SNP	Primer Sequence (5′ to 3′)	Restriction Enzyme	Annealing Temperature	Expected Amplicons	Reference
C-158T (rs7482144)	Forward primer: GAACTTAAGAGATAATGGCCTAA Reverse primer: ATGACCCATGGCGTCTGGACTAG	*Xmn*I	60 °C	Wild-type (C/C)—641 bp; Heterozygous (C/T)—641 bp, 418 bp, and 223 bp; Variant (T/T)—418 bp and 223 bp	[18]
G79A (rs33950507)	Forward primer: CATTTGCTTCTGACACAACTG Reverse primer: TTGAGGTTGTCCAGGTAG	*Mnl*I	50 °C	Wild-type (G/G)—171 bp, 135 bp, 62 bp, and 59 bp; Heterozygous (G/A)—233 bp, 171 bp, 135 bp, 62 bp, and 59 bp; Variant (A/A)—233 bp, 135 bp, and 59 bp	[19]
C16G (rs10768683)	Forward primer: TTAGGCTGCTGGTGGTC Reverse primer: CAATCATTCGTCTGTTTCC	*Ava*II	58 °C	Wild-type (C/C)—320 bp and 25 bp; Heterozygous (C/G)—320 bp, 214 bp, 106 bp, and 25 bp; Variant (G/G)—214 bp, 106 bp, and 25 bp	[20]
C-551T	Forward primer: CTTTGGGTTGTAAGTGA Reverse primer: TTGGGATATGTAGATGG	*Rsa*I	60 °C	Wild-type (C/C)—386 bp; Heterozygous (C/T)—386 bp, 219 bp, and 167 bp; Variant (T/T)—219 bp and 167 bp	[20]

**Table 2:** Association of the β-like globin C-158T SNP with malaria risk.

SNP	Cases	Controls	OR (95% CI)	*p*-Value
Overall				
Allele				
C	361	313	1.00 (Reference)	-
T	15	27	0.48 (0.25–0.92)	0.027 *
Genotype				
CC	173	145	1.00 (Reference)	-
CT	15	23	0.55 (0.28–1.09)	0.085
TT	0	2	-	-
CT + TT	15	25	0.50 (0.26–0.99)	0.047 *
				
*P. knowlesi*				
Allele				
C	258	313	1.00 (Reference)	-
T	10	27	0.45 (0.21–0.95)	0.035 *
Genotype				
CC	124	145	1.00 (Reference)	-
CT	10	23	0.51 (0.23–1.11)	0.089
TT	0	2	-	-
CT + TT	10	25	0.47 (0.22–1.01)	0.054
				
*P. falciparum*				
Allele				
C	54	313	1.00 (Reference)	-
T	2	27	0.43 (0.10–1.86)	0.258
Genotype				
CC	26	145	1.00 (Reference)	-
CT	2	23	0.48 (0.11–2.18)	0.346
TT	0	2	-	-
CT + TT	2	25	0.45 (0.10–2.00)	0.292
				
*P. vivax*				
Allele				
C	49	313	1.00 (Reference)	-
T	3	27	0.71 (0.21–2.43)	0.585
Genotype				
CC	23	145	1.00 (Reference)	-
CT	3	23	0.82 (0.23–2.96)	0.765
TT	0	2	-	-
CT + TT	3	25	0.76 (0.21–2.71)	0.668

* Statistically significant.

**Table 3:** Association of the β-like globin G79A SNP with malaria risk.

SNP	Cases	Controls	OR (95% CI)	*p*-Value
Overall				
Allele				
G	372	337	1.00 (Reference)	-
A	4	3	1.21 (0.27–5.44)	0.806
Genotype				
GG	184	167	1.00 (Reference)	-
GA	4	3	1.21 (0.27–5.49)	0.805
AA	0	0	-	-
GA + AA	4	3	1.21 (0.27–5.49)	0.805
				
*P. knowlesi*				
Allele				
G	268	337	1.00 (Reference)	-
A	0	3	-	-
Genotype				
GG	134	167	1.00 (Reference)	-
GA	0	3	-	-
AA	0	0	-	-
GA + AA	0	3	-	-
				
*P. falciparum*				
Allele				
G	53	337	1.00 (Reference)	-
A	3	3	6.36 (1.25–32.33)	0.026 *
Genotype				
GG	25	167	1.00 (Reference)	-
GA	3	3	6.68 (1.28–34.94)	0.025 *
AA	0	0	-	-
GA + AA	3	3	6.68 (1.28–34.94)	0.025 *
				
*P. vivax*				
Allele				
G	51	337	1.00 (Reference)	-
A	1	3	2.20 (0.22–21.58)	0.498
Genotype				
GG	25	167	1.00 (Reference)	-
GA	1	3	2.23 (0.22–22.25)	0.496
AA	0	0	-	-
GA + AA	1	3	2.23 (0.22–22.25)	0.496

* Statistically significant.

**Table 4:** Association of the β-like globin C16G SNP with malaria risk.

SNP	Cases	Controls	OR (95% CI)	*p*-Value
Overall				
Allele				
C	214	181	1.00 (Reference)	-
G	162	159	0.86 (0.64–1.16)	0.323
Genotype				
CC	65	56	1.00 (Reference)	-
CG	84	69	1.05 (0.65–1.69)	0.845
GG	39	45	0.75 (0.43–1.30)	0.305
CG + GG	123	114	0.93 (0.60–1.44)	0.744
				
*P. knowlesi*				
Allele				
C	144	181	1.00 (Reference)	-
G	124	159	0.98 (0.71–1.35)	0.903
Genotype				
CC	43	56	1.00 (Reference)	-
CG	58	69	1.09 (0.65–1.86)	0.737
GG	33	45	0.96 (0.52–1.74)	0.881
CG + GG	91	114	1.04 (0.64–1.69)	0.875
				
*P. falciparum*				
Allele				
C	33	181	1.00 (Reference)	-
G	23	159	0.79 (0.45–1.41)	0.429
Genotype				
CC	9	56	1.00 (Reference)	-
CG	15	69	1.35 (0.55–3.32)	0.510
GG	4	45	0.55 (0.16–1.91)	0.350
CG + GG	19	114	1.04 (0.44–2.44)	0.934
				
*P. vivax*				
Allele				
C	37	181	1.00 (Reference)	-
G	15	159	0.46 (0.24–0.87)	0.017 *
Genotype				
CC	13	56	1.00 (Reference)	-
CG	11	69	0.69 (0.29–1.65)	0.401
GG	2	45	0.19 (0.04–0.89)	0.035 *
CG + GG	13	114	0.49 (0.21–1.13)	0.094

* Statistically significant.

**Table 5:** Association of the β-like globin C-551T SNP with malaria risk.

SNP	Cases	Controls	OR (95% CI)	*p*-Value
Overall				
Allele				
C	205	161	1.00 (Reference)	-
T	171	179	0.75 (0.56–1.01)	0.056
Genotype				
CC	62	51	1.00 (Reference)	-
CT	81	59	1.13 (0.69–1.86)	0.634
TT	45	60	0.62 (0.36–1.05)	0.077
CT + TT	126	119	0.87 (0.56–1.36)	0.545
				
*P. knowlesi*				
Allele				
C	145	161	1.00 (Reference)	-
T	123	179	0.76 (0.55–1.05)	0.099
Genotype				
CC	47	51	1.00 (Reference)	-
CT	51	59	0.94 (0.54–1.62)	0.818
TT	36	60	0.65 (0.37–1.15)	0.142
CT + TT	87	119	0.79 (0.49–1.29)	0.348
				
*P. falciparum*				
Allele				
C	30	161	1.00 (Reference)	-
T	26	179	0.78 (0.44–1.37)	0.389
Genotype				
CC	9	51	1.00 (Reference)	-
CT	12	59	1.15 (0.45–2.96)	0.768
TT	7	60	0.66 (0.23–1.90)	0.442
CT + TT	19	119	0.90 (0.38–2.13)	0.819
				
*P. vivax*				
Allele				
C	30	161	1.00 (Reference)	-
T	22	179	0.66 (0.37–1.19)	0.167
Genotype				
CC	6	51	1.00 (Reference)	-
CT	18	59	2.59 (0.96–7.03)	0.061
TT	2	60	0.28 (0.05–1.47)	0.133
CT + TT	20	119	1.43 (0.54–3.77)	0.471

**Table 6:** Linkage disequilibrium analysis of the β-like globin SNPs.

SNP	C-158T	G79A	C16G	C-551T
C-158T		1.000	0.402	0.477
G79A	0.001		0.238	0.657
C16G	0.012	0.000		0.698
C-551T	0.015	0.004	0.414	

Note: Values in the upper and lower diagonal indicated the D’ and r^2^ for the SNP combinations, respectively.

**Table 7:** Haplotype analysis of the β-like globin SNPs.

Haplotype ^#^	Cases	Control	OR (95% CI)	*p*-Value
CGCC	0.524	0.300	2.51 (1.84–3.42)	<0.001 *
CGCT	0.035	0.174	0.17 (0.09–0.31)	<0.001 *
CGGC	0.011	0.131	0.07 (0.03–0.20)	<0.001 *
CGGT	0.380	0.307	1.35 (0.99–1.84)	0.061
TGGT	0.040	0.022	1.77 (0.73–4.29)	0.199

^#^ Based on the physical marker order: C-158T, G79A, C16G, and C-551T. * Statistically significant.

## Data Availability

The data of this study are included in tables, figures, and referenced articles.

## Supplementary Materials

The following supporting information can be downloaded at: https://www.mdpi.com/article/10.3390/genes13071229/s1, Figure S1: Restriction enzyme-digested fragments of the β-like globin SNPs that were analyzed in agarose gel.
