# Supplementary material for: Risk Association, Linkage Disequilibrium, and Haplotype Analyses of β-Like Globin Gene Polymorphisms with Malaria Risk in the Sabah Population of Malaysian Borneo
Source: Genes (Basel). 2022 Jul 11;13(7):1229. doi: 10.3390/genes13071229 (PMC9319382; doi:10.3390/genes13071229)
Supplement: Supplementary file 1 [file genes-13-01229-s001.zip › genes-1745580-supplementary.pdf]

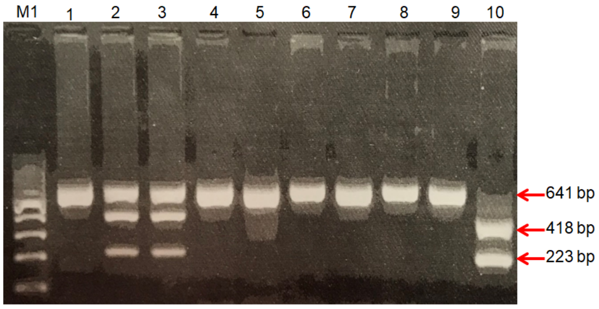

(**a**)

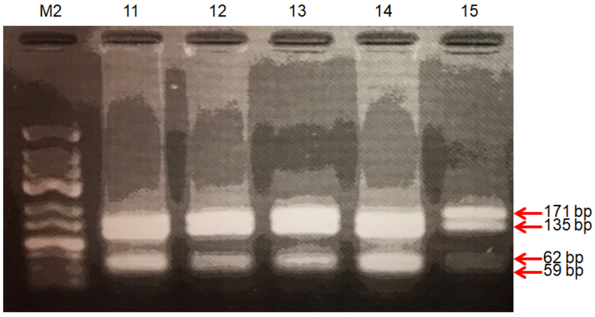

(**b**)

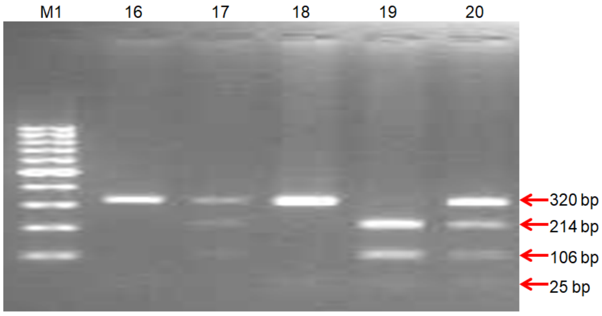

(**c**)

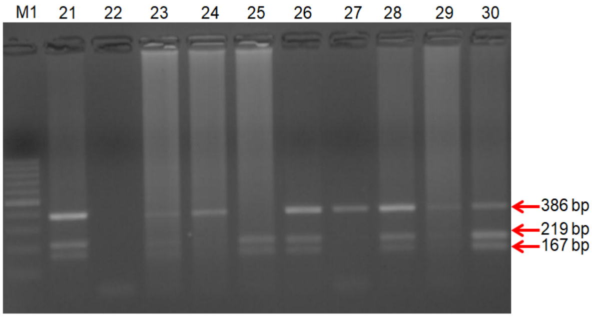

(**d**)

Figure S1. Restriction enzyme-digested fragments of the β-like globin SNPs that were analyzed in agarose gel. (**a**) A representative gel picture of the *Xmn*I-digested fragments for the β-like globin C-158T SNP. Well no. 1 and 4-9 show the homozygous wild-type (C/C) genotype, well no. 2 and 3 show the heterozygous (C/T) genotype, while well no. 10 shows the homozygous variant (T/T) genotype; (**b**) A representative gel picture of the *Mnl*I-digested fragments for the β-like globin G79A SNP. Well no. 11-15 show the homozygous wild-type (G/G) genotype; (**c**) A representative gel picture of the *Ava*II-digested fragments for the β-like globin C16G SNP. Well no. 16 and 18 show the homozygous wild-type (C/C) genotype, well no. 17 and 20 show the heterozygous (C/G) genotype, while well no. 19 shows the homozygous variant (G/G) genotype; (**d**) A representative gel picture of the *Rsa*I-digested fragments for the β-like globin C-551T SNP. Well no. 27 shows the homozygous wild-type (C/C) genotype, well no. 21, 23-24, 26, 28-30 show the heterozygous (C/T) genotype, well no. 25 shows the homozygous variant (T/T) genotype, while well no. 22 shows the absence of digested fragments. M1: GeneRuler™ 100 bp DNA Ladder; M2: GeneRuler™ Low Range DNA Ladder.
